# Supplementary material for: Editing an α-globin enhancer in primary human hematopoietic stem cells as a treatment for β-thalassemia
Source: Nat Commun. 2017 Sep 4;8:424. doi: 10.1038/s41467-017-00479-7 (PMC5583283; doi:10.1038/s41467-017-00479-7)
Supplement: Supplementary file 1 — Supplementary Information [file 41467_2017_479_MOESM1_ESM.pdf]

File name: Supplementary Information
Description: Supplementary figures and supplementary tables.

File name: Peer review file
Description:

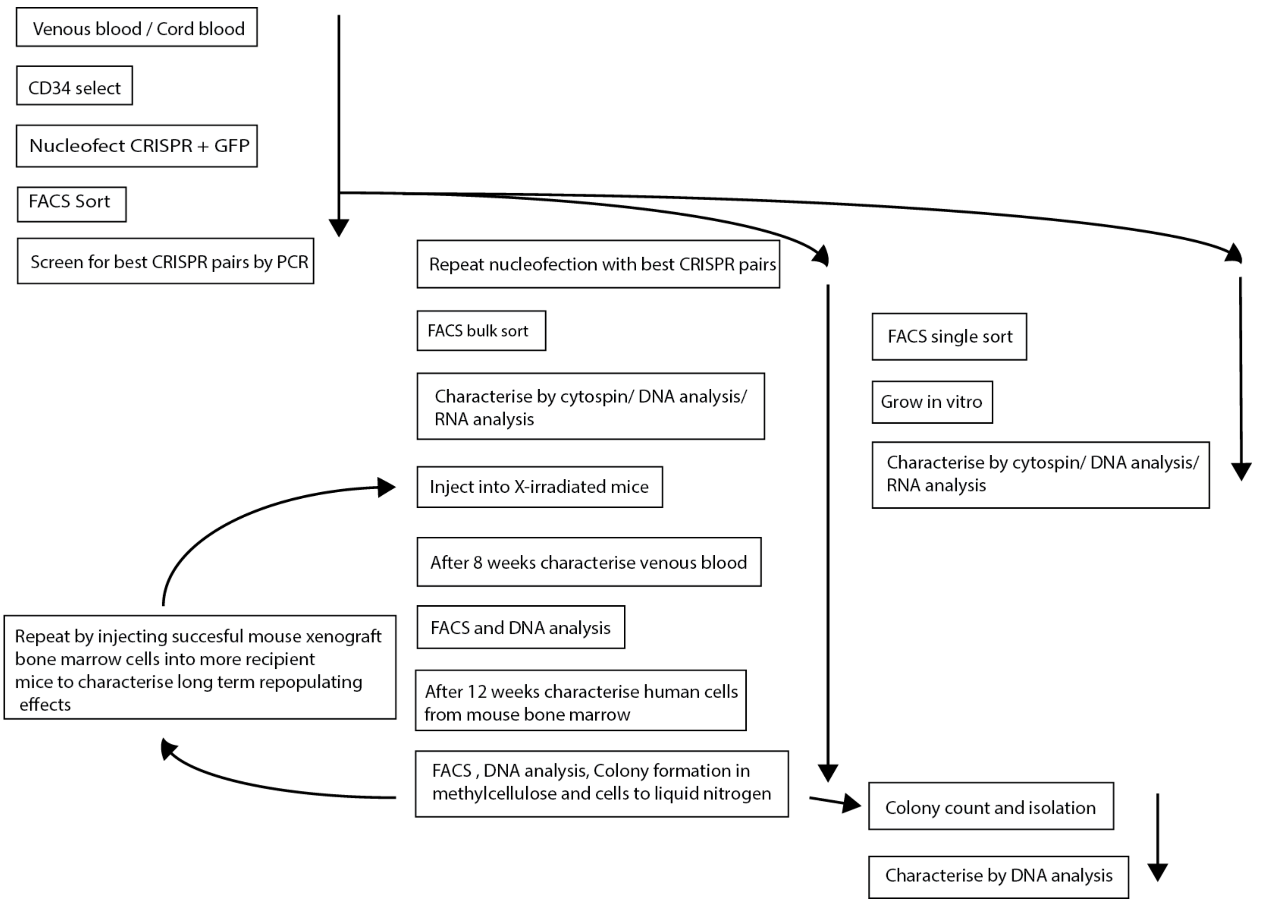

Supplementary figure 1 – Flow diagram summarizing the overall experimental strategy

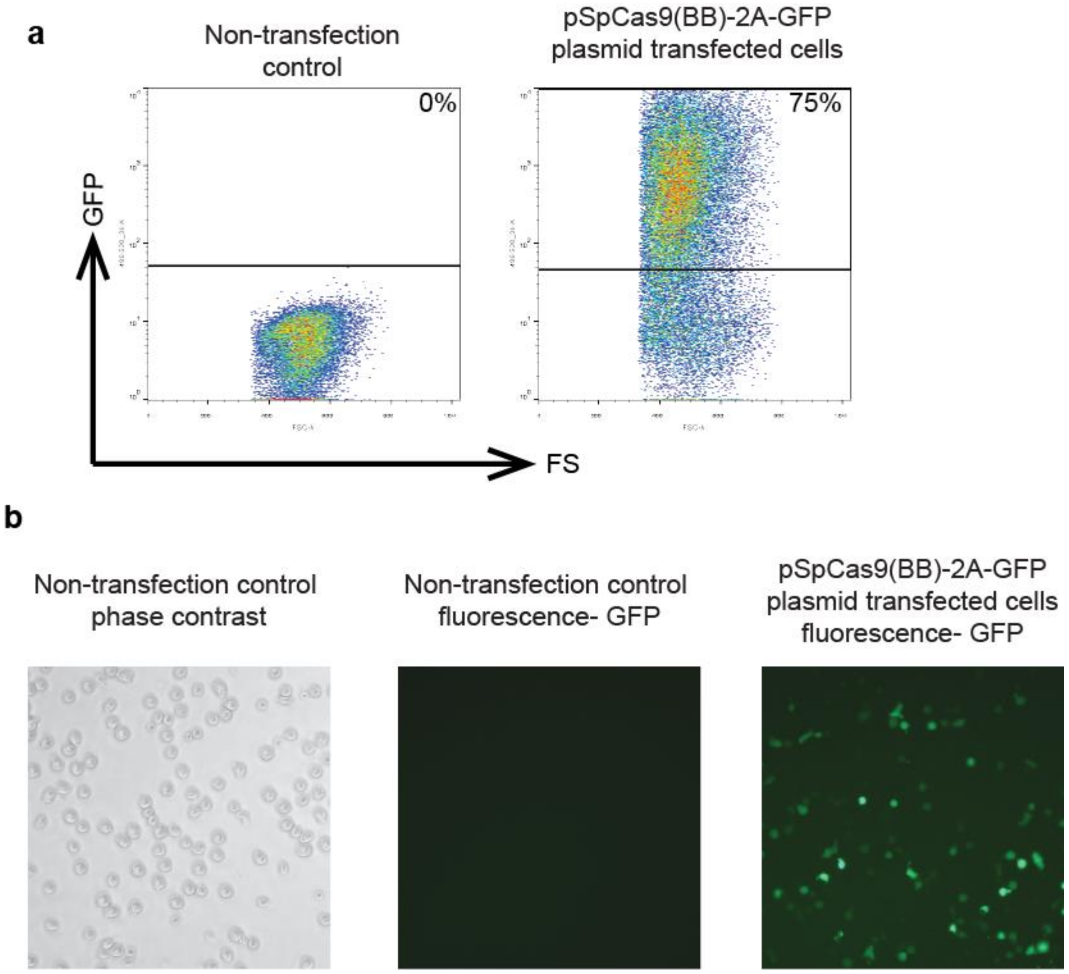

Supplementary figure 2 - Efficiency of the transfection of primary CD34+ HSPCs. (a) Flow cytometry plots of non-transfection control and CRISPR plasmid transfected cells demonstrating GFP expression. (b) Fluorescent microscopy image of non-transfection control and CRISPR plasmid transfected cells showing green fluorescence in transfected cells. Exposure times of fluorescence images of control and plasmid transfected cells were constant.

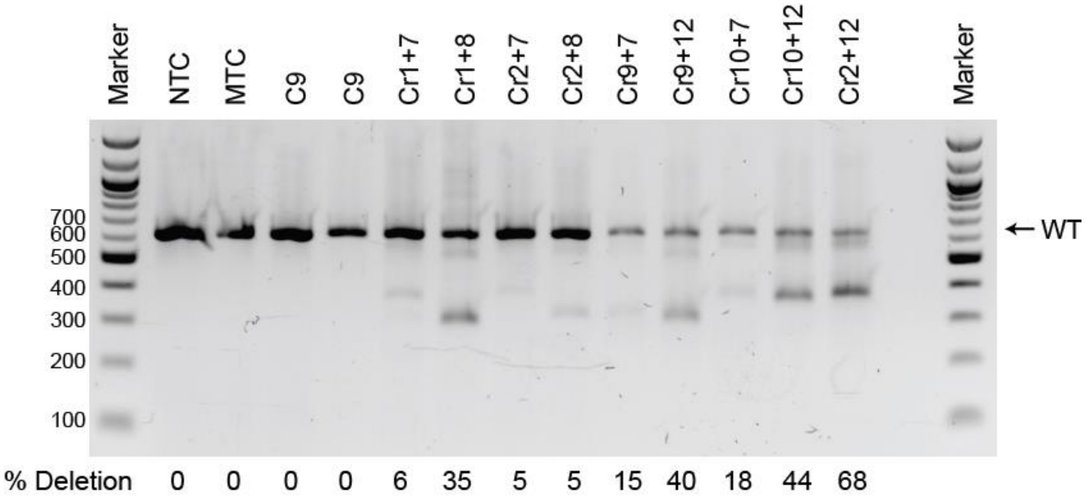

Supplementary figure 3 - Gel electrophoresis image of CD34+ HSPCs transfected with different CRISPR/Cas9 plasmid pairs analyzed by PCR. Wild type (WT) amplicon is 613 bp and depending on the sgRNA target site, deletions produced amplicons with lengths between 302 – 390 bp. Percentages of mutated alleles determined by band intensity is shown below each lane. Abbreviations: NTC, non-transfection control; MTC, mock transfection control; C9, Cas9-only control.

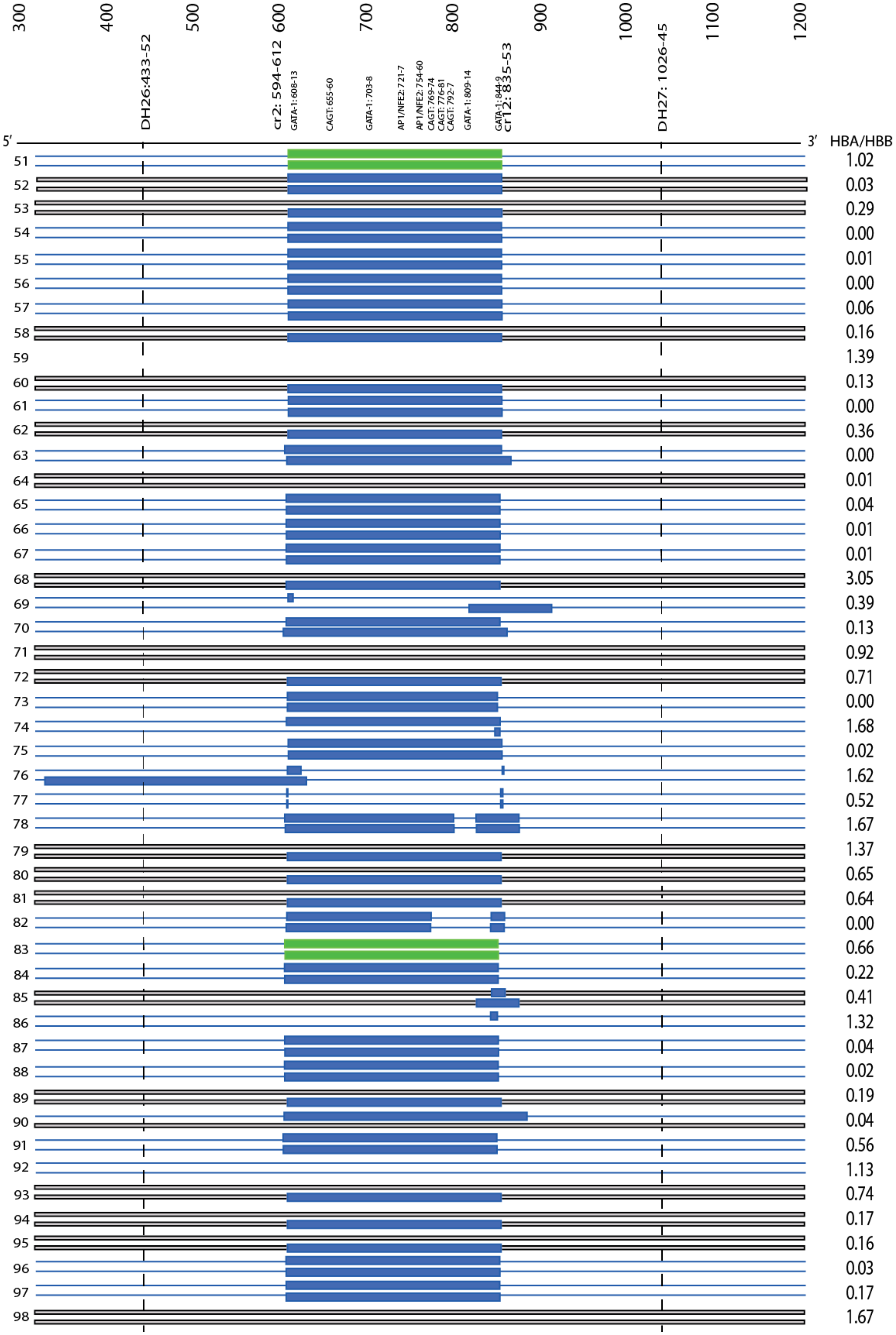

300
400
500
600
700
800
900
1000
1100
1200
DH26:433-52
cr2: 594-612
GATA-1: 608-13
CAGT: 655-60
GATA-1: 703-8
AP1/NFE2: 721-7
AP1/NFE2: 754-60
CAGT: 769-74
CAGT: 776-81
CAGT: 792-7
GATA-1: 809-14
GATA-1: 844-9
cr12: 835-53
DH27: 1026-45
5′
3′
HBA/HBB
51 1.02
52 0.03
53 0.29
54 0.00
55 0.01
56 0.00
57 0.06
58 0.16
59 1.39
60 0.13
61 0.00
62 0.36
63 0.00
64 0.01
65 0.04
66 0.01
67 0.01
68 3.05
69 0.39
70 0.13
71 0.92
72 0.71
73 0.00
74 1.68
75 0.02
76 1.62
77 0.52
78 1.67
79 1.37
80 0.65
81 0.64
82 0.00
83 0.66
84 0.22
85 0.41
86 1.32
87 0.04
88 0.02
89 0.19
90 0.04
91 0.56
92 1.13
93 0.74
94 0.17
95 0.16
96 0.03
97 0.17
98 1.67

Supplementary figure 4 – Genotype analysis by sequencing of individual single cell clones edited using CRISPR pair Cr2+Cr12 (Clone 51-98). Two blue lines represent each allele and different mutations are represented as follows: blue bars – deletions, green bars – inversions. Single base pair variations that were observed around sgRNA binding sites are not shown. The grey bars extending across represent sequence data which are not available either because not attempted (non-deleted clones by PCR were not sequenced) or unreadable. Vertical dashed lines show the sites of forward (DH26) and reverse (DH27) primers used for PCR amplification shown in figure 3a. Expected break points of CRISPR 2 (Cr2) and CRISPR 12 (Cr12) as well as the important transcription factor binding motifs of the MCS-R2 enhancer are annotated at the top of the diagram. The α/β-globin mRNA ratios (HBA/HBB) of individual clones are presented next to the genotype diagram. The co-orodinates from this contig are from Hum Mar 2006 (NCBI36/hg18) Assembly where 1 on this contig is equivalent to chr16: 102901. Deletions that extended beyond the oligonucleotides DH26 and/or DH27 were analysed with oligos further out.

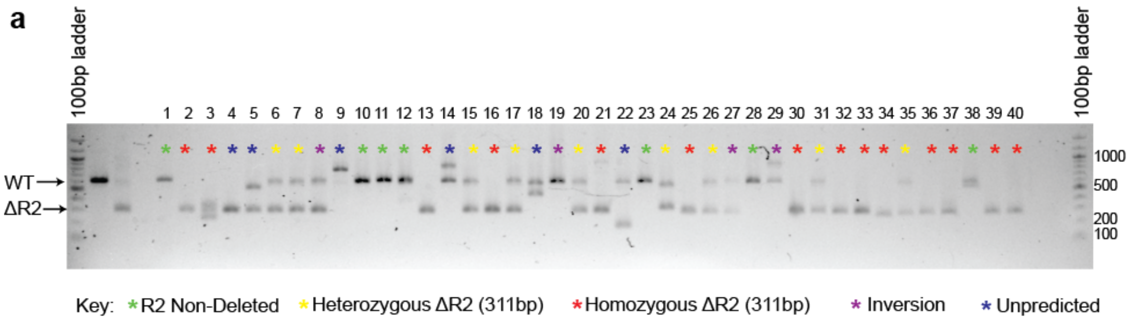

b

Mutation type	Frequency (%) (N=40)
R2 Non-Deleted	7 (18%)
Heterozygous ΔR2 (311bp)	9 (22%)
Homozygous ΔR2 (311bp)	14 (35%)
Inversions	4 (10%)
Unpredicted	6 (15%)

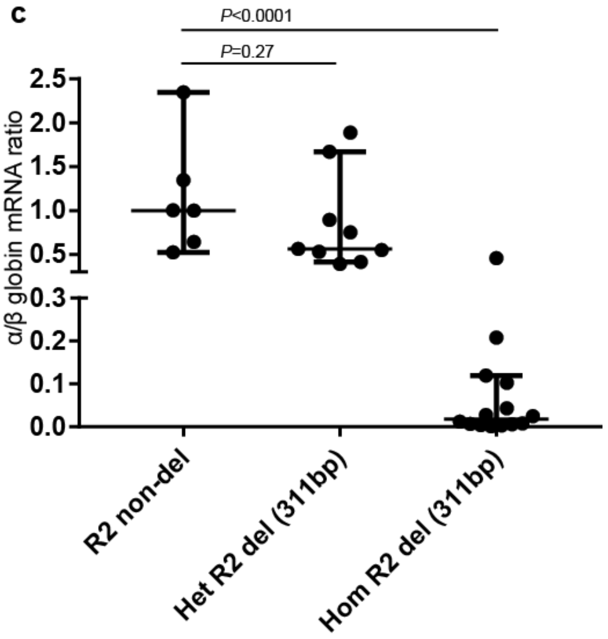

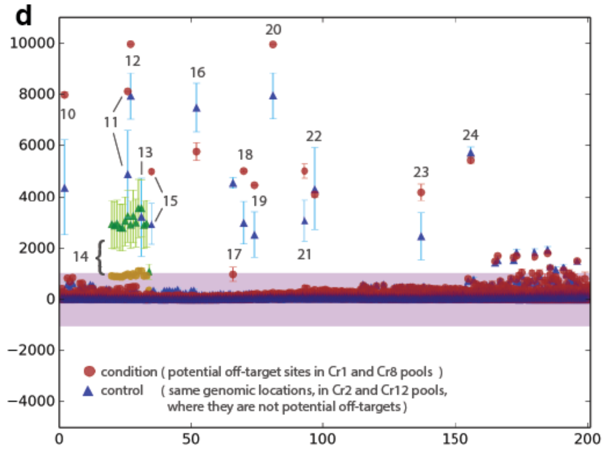

Supplementary figure 5 – Single cell clone analysis of targeted deletion of MCS-R2 using CRISPR pair Cr1+Cr8. (a) Gel electrophoresis image of genomic DNA from 40 individual single

cell clones (from 3 independent donors) analyzed by PCR. The amplicon from the wild type allele is 613bp and the mutated amplicon is 302bp. Clones are numbered 1 – 40. Extended genotype analysis of these clones by sequencing is presented in supplementary figure 6. (b) Frequency of different types of mutations generated. (c) α/β-globin mRNA ratios of individual clones of erythroid cells which are non-deleted (R2 non-del) (n = 7) and heterozygous (Het R2 del.) (n = 9) or homozygous (Hom R2 del.) (n = 14) for a 311bp deletion of MCS-R2 region analyzed by qPCR; median (horizontal bar) and 95% confidence interval (error bar) are shown and *P* values were calculated using Mann-Whitney test. (d) Meta-plot of all off-target loci for CRISPR pair Cr1 and Cr8. All captured sites are plotted on the same x-axis, showing +/- 100 bases from each potential off-target site. Counts deviating from the reference sequence which are normalized to 10,000 counts are plotted in the y-axis. The values are means of the libraries where each library is a pool of five independent clones. The shaded violet area denotes +/- 1000 counts and only data over this threshold was considered as off-target. Error bars represent standard error of the mean (SEM) for each base at each locus. Potential off-target hits for Cr1 and Cr8 (condition) are plotted alongside those of a control group (control). The numbers are annotated in supplementary table 4. All of the variations from the reference sequence were shown to be known SNPs or indels or novel variations common to both control and condition and therefore unrelated to potential off-target activity.

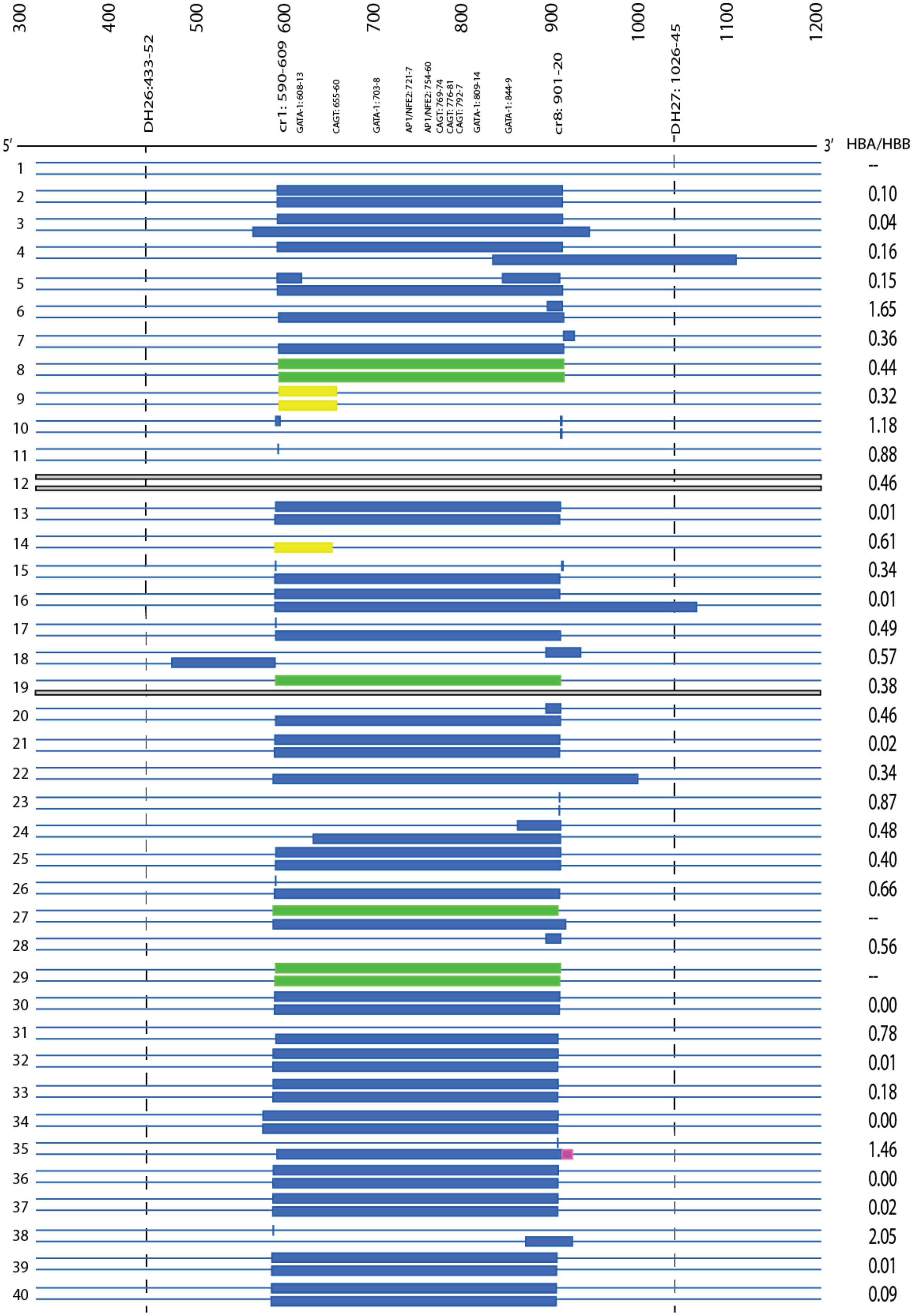

300
400
500
600
700
800
900
1000
1100
1200
DH26:433-52
cr1: 590-609
GATA-1:608-13
CAGT:655-60
GATA-1: 703-8
AP1/NFE2:721-7
AP1/NFE2: 754-60
CAGT: 769-74
CAGT: 776-81
CAGT: 792-7
GATA-1: 809-14
GATA-1: 844-9
cr8: 901-20
DH27: 1026-45
5′
3′
HBA/HBB
1 --
2 0.10
3 0.04
4 0.16
5 0.15
6 1.65
7 0.36
8 0.44
9 0.32
10 1.18
11 0.88
12 0.46
13 0.01
14 0.61
15 0.34
16 0.01
17 0.49
18 0.57
19 0.38
20 0.46
21 0.02
22 0.34
23 0.87
24 0.48
25 0.40
26 0.66
27 --
28 0.56
29 --
30 0.00
31 0.78
32 0.01
33 0.18
34 0.00
35 1.46
36 0.00
37 0.02
38 2.05
39 0.01
40 0.09

Supplementary figure 6 – Genotype analysis by sequencing of individual single cell clones edited using CRISPR pair Cr1+Cr8 (Clone 1-40). Two blue lines represent each allele and different mutations are represented as follows: blue bars – deletions, green bars – inversions, yellow bar – vector insertion, pink bar – insertion. Single base pair variations that were observed around gRNA binding sites are not shown. The grey bars extending across represent lack of sequence data (either not attempted or unreadable). Vertical dashed lines show the sites of forward (DH26) and reverse (DH27) primers used for PCR amplification shown in supplementary figure 5a. Expected break points of CRISPR 1 (Cr1) and CRISPR 8 (Cr8) as well as the important transcription factor binding motifs of the MCS-R2 enhancer are annotated at the top of the diagram. The α/β-globin mRNA ratios (HBA/HBB) of individual clones are presented next to the genotype diagram. The co-orodinates from this contig are from Hum Mar 2006 (NCBI36/hg18) Assembly where 1 on this contig is equivalent to chr16: 102901. Deletions that extended beyond the oligonucleotides DH26 and/or DH27 were analysed with oligos further out.

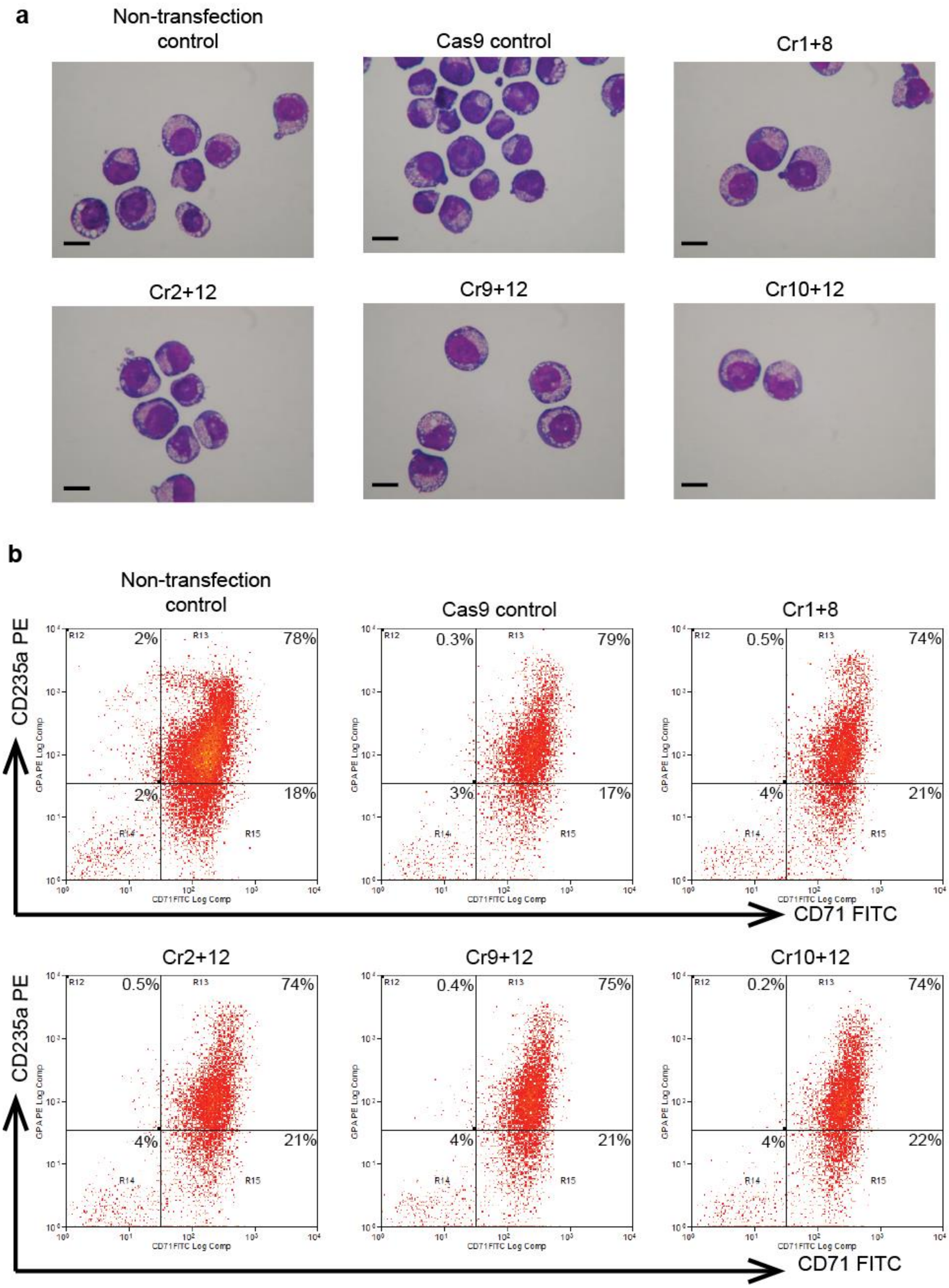
a
Non-transfection control
Cas9 control
Cr1+8
Cr2+12
Cr9+12
Cr10+12
b
Non-transfection control
Cas9 control
Cr1+8
CD235a PE
CD71 FITC
2%
78%
2%
18%
0.3%
79%
3%
17%
0.5%
74%
4%
21%
Cr2+12
Cr9+12
Cr10+12
0.5%
74%
4%
21%
0.4%
75%
4%
21%
0.2%
74%
4%
22%

Supplementary figure 7 – Erythroid differentiation of CRISPR edited cells. (a) Representative cytospins of cells stained by modified Wright stain demonstrating cells of similar differentiation stages in genome edited and control samples; scale bar represents 10 µm (n=3) (b) Flow cytometry plots of cells stained with FITC-conjugated anti-CD71 and PE-conjugated anti-CD235a antibodies demonstrating the expected erythroid differentiation profile for genome-edited and control cells (n=3). Gating strategy is shown in supplementary figure 10.

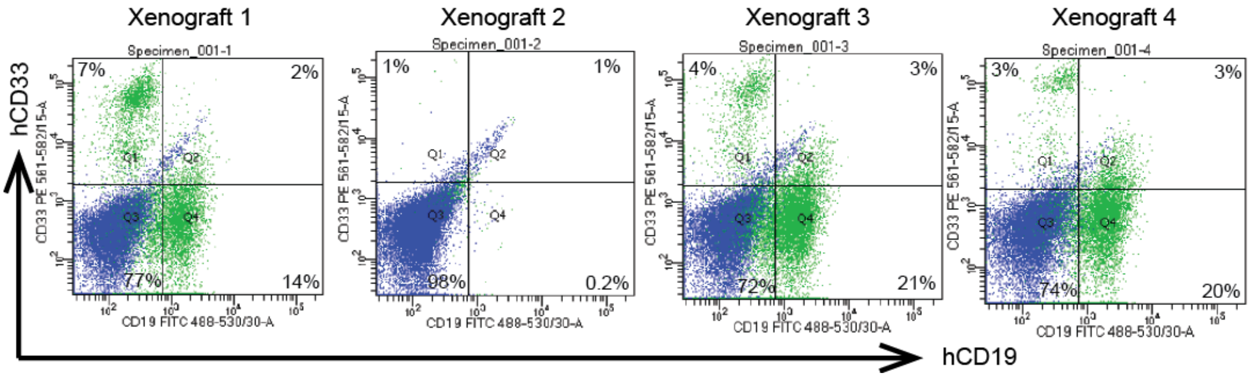

Supplementary figure 8 – Multipotentiality of genome edited human HSCs from xenograft mice. Flow cytometry plots of harvested bone marrow from xenograft mice gated for live human CD45+ cells demonstrating expression of human CD19 (lymphoid) and human CD33 (myeloid) cell surface markers. Gating strategy is shown in supplementary figure 10.

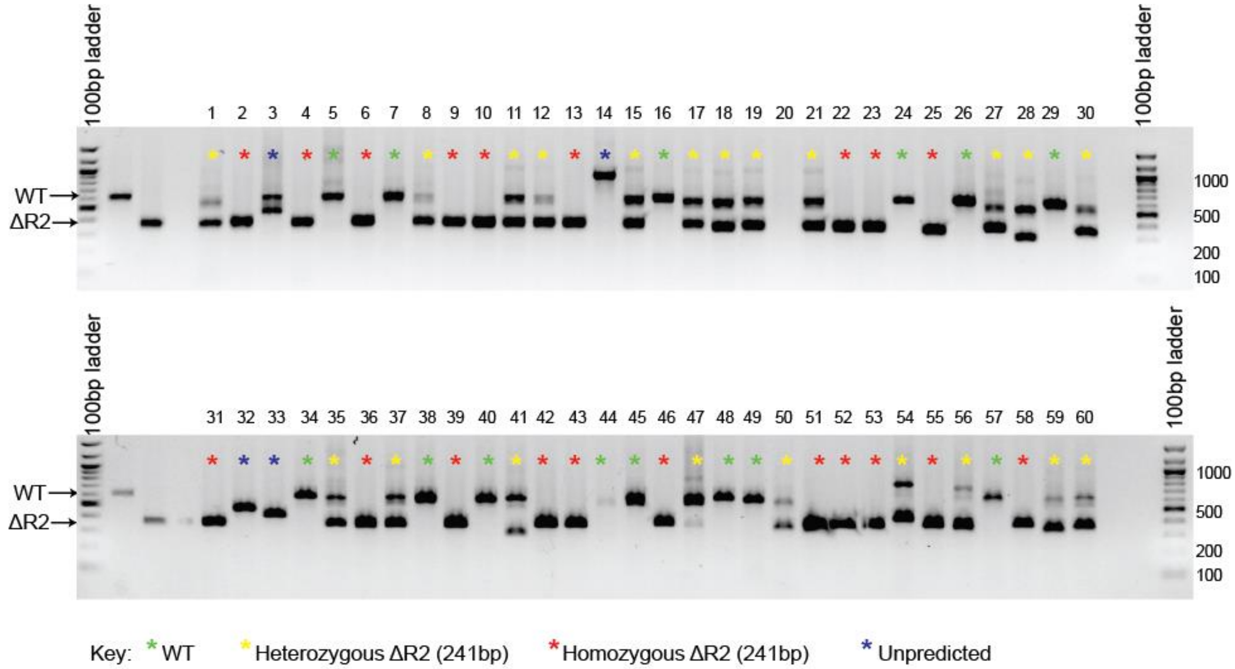

Supplementary figure 9 – Single cell clone analysis of targeted deletion of MCS-R2 in HbE β-thalassemia cells using CRISPR pair Cr2+Cr12. Gel electrophoresis image of genomic DNA from 60 individual single cell clones (from 3 independent donors) analyzed by PCR. The amplicon from the wild type allele is 613bp and the mutated amplicon is 372bp. Clones are numbered 1 – 60.

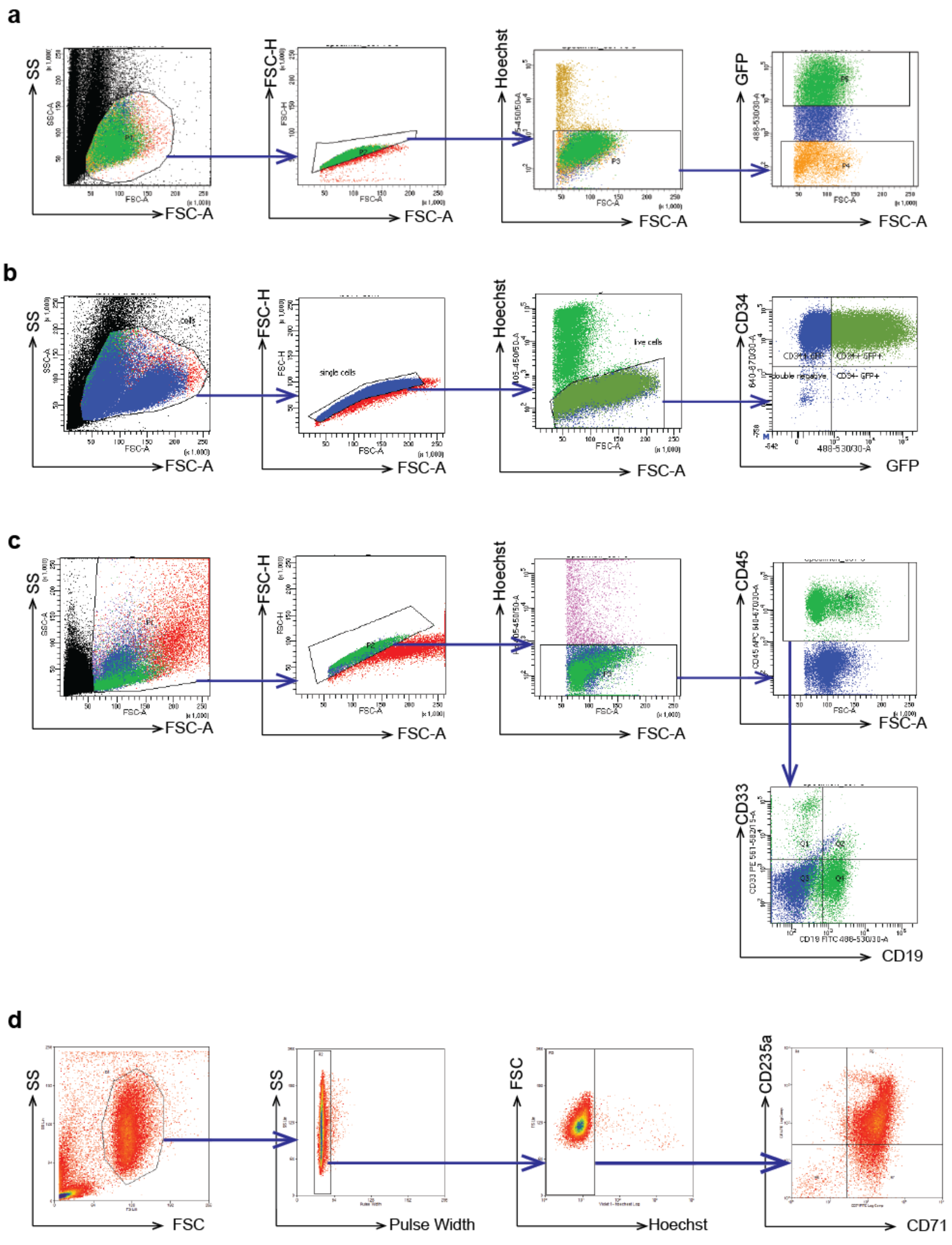
a
SS
FSC-A
FSC-H
FSC-A
Hoechst
FSC-A
GFP
FSC-A
b
SS
cells
FSC-A
FSC-H
single cells
FSC-A
Hoechst
live cells
FSC-A
CD34
double negative
CD34+ GFP+
GFP
c
SS
FSC-A
FSC-H
FSC-A
Hoechst
FSC-A
CD45
FSC-A
CD33
CD19
d
SS
FSC
SS
Pulse Width
FSC
Hoechst
CD235a
CD71

Supplementary figure 10 – Flow cytometry gating strategy. (a) Gating strategy for figure 2b. (b) Gating strategy for figure 4b. (c) Gating strategy for figure 4c and supplementary figure 8. (d) Gating strategy for supplementary figure 7b. Abbreviations: FSC, Forward Scatter; SS, Side Scatter; GFP, Green Fluorescent Protein.

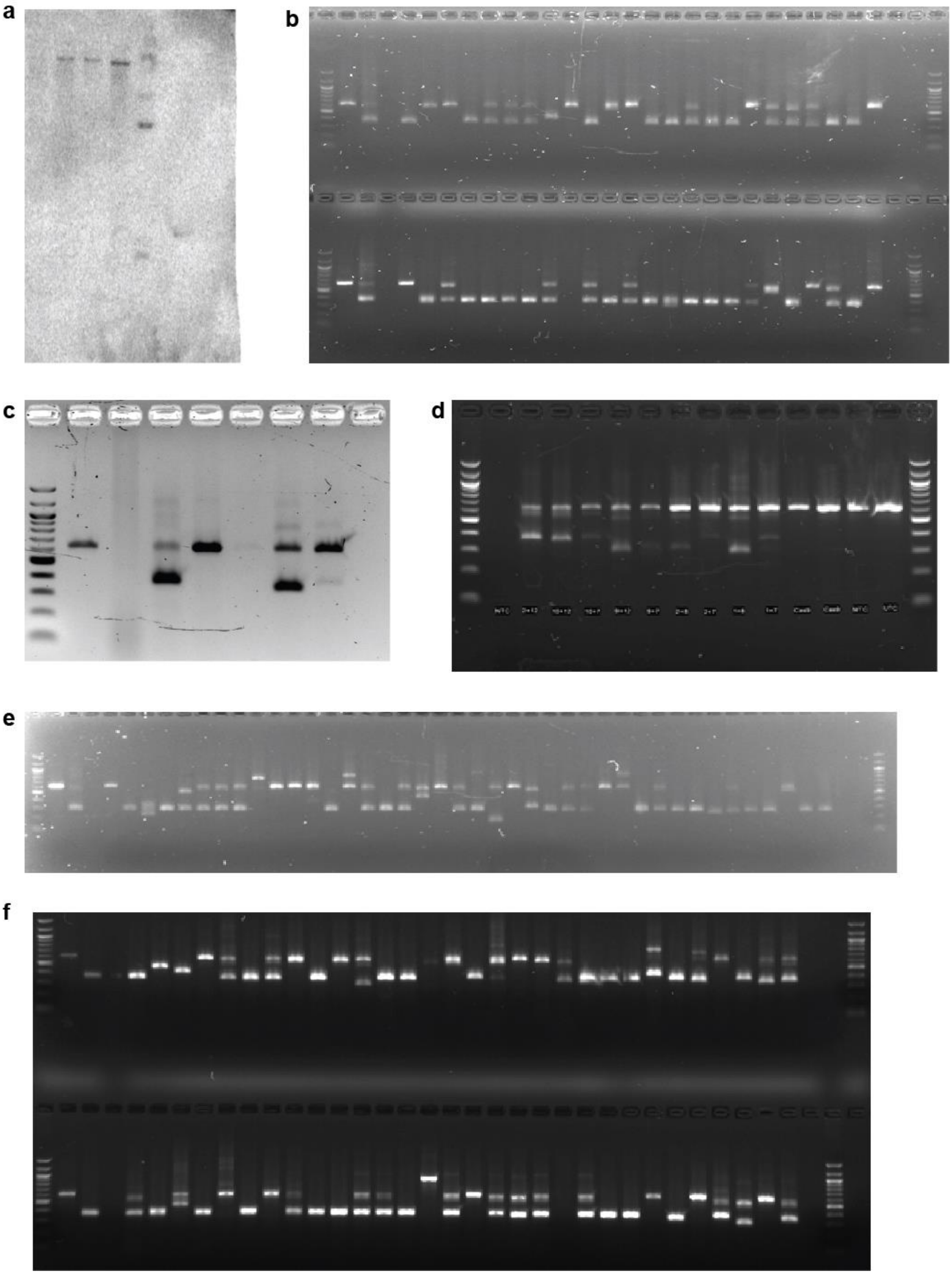
a
b
c
d
e
f

Supplementary figure 11 - Uncropped scans of all gels. (a) Uncropped image of figure 1e. (b) Uncropped image of figure 3a. (c) Uncropped image of figure 4d. (d) Uncropped image of supplementary figure 3. (e) Uncropped image of supplementary figure 5. (f) Uncropped image of supplementary figure 9.

Supplementary table 1 – Clinical and molecular characteristics of individuals with natural mutation [$(\alpha\alpha)^{ALT}$] confined to MCS-R2

	MC	RC
Age	59 years	18 years
Hemoglobin HPLC		
HbA_2	1.4%	2.7%
HbF	0.3%	0.5%
HbH	~3%	Not detected
Serum Iron (μg/dL)	108 (Normal 59-158)	80 (Normal 37-145)
Serum Transferrin (mg/dL)	210 (Normal 200-360)	295 (Normal 200-360)
Serum Ferritin (ng/ml)	1139 (Normal 28-397)	28 (Normal 6-159)
β-globin genotype (sequencing)	Normal β-globin genes	Normal β-globin genes
α-globin expression level relative to a normal control	0.3	0.55

Supplementary table 2 - sgRNA sequences of CRISPR target sites within and around MCS-R2 enhancer region

CRISPR ID	Target sequence
Cr1	TCGACCCTCTGGAACCTAT
Cr2	CGACCCTCTGGAACCTATC
Cr7	CTCCTGTTTATCTGAGAGG
Cr8	GACCCAGACAGTAAATACG
Cr9	CTTCTGCAACCATGATGAC
Cr10	AGAGGGGCCCTCGACCCTC
Cr12	CCCTCCTGTTTATCTGAGA

Supplementary table 3 - off-target sites and probe designs

sgRNA	Location	Sequence	Mismatches	Strand	Type	Gene	Upstream Probe location	Upstream Probe seq	Downstream Probe location	Downstream Probe seq
2	16:113495-113516	CGACCCTCTGGAACCTATC NGG	0	+	On-target	MCS-R2	-	-	-	-
2	22:41697129-41697151	TGAACCCTCTGGAACCTCTC TGG	4	-	Exonic	C22orf46	chr22:41697078-41697128	AGCAGGAAAGGTATTGTGAGGCCCAAGGCTCCCCACCAGGCCTTAAGTCC	chr22:41697151-41697201	GCGCCCAGACAGAAAAAGCTCGAAACTTCCTATACAAGCCAGAGCTCTGG
2	12:132908486-132908508	CCGCCCCTCTGGGACCCATC CGG	4	-	Intronic	CHFR	chr12:132908435-132908485	ACGCCTAAGACTGCGCGCCGCAGCCCGCCCTGTCTAGCTGAGGCGCAGAC	chr12:132908508-132908558	GTCGGGGCGGAGAGCCGCGCCCAAAGGCAATGGGAGCCGCACGCTGCTAG
2	X:123429476-123429498	AAGACCCTCTGGAACTAATC TGG	4	+	Intronic	GRIA3	chrX:123429425-123429475	ACCAGATCATAATCCCCAACTGGGAGGAGGGGTCCAATGTTGGTCCAATG	chrX:123429498-123429548	CCTTGACCCTCACTACTATGGCAGTCCCAGAAGCTTATTTAGAAACAATT
2	1:18294783-18294805	CAGACCCTCTGGAGCCTAAC GGG	4	+	Intronic	IGSF21	chr1:18294712-18294762	CAAGCAAAGAACACCCCAGGGAGCTGAGCCGTCCTGTTTGGTGACCACCA	chr1:18294805-18294855	ATCACGGAGGATTGTCAGCCCTCCCAACCAGCCATGCTGGGGGCGACTGC
2	14:104936545-104936567	TCACCCCTCTGGAACCTCTC AGG	4	+	Exonic	PLD4	chr14:104936454-104936504	CCCCAACTCAGCACAGGGAACATATGCCCATGGTGGCATGGGGAAAGAAC	chr14:104936567-104936617	GCAGCACGAGTGGTAGCCTCGGAGTGGCCTTCACCCTCCAGGCACTGCGG
2	5:139259569-139259591	ATGACCCTCTGGAACCCCTC TGG	4	+	Intronic	SIL1	chr5:139259478-139259528	CCAGGCCCTACTTTACTTCCTTGGACATTGTTTGCTCTTGTGTTCTGGGT	-	-
2	4:86840217-86840239	CCGACACTCTGGATTCTATC AGG	4	+	Intronic	SLC10A6	-	-	chr4:86840279-86840329	CCTCTCTAAACAATCTTGCTTGTCAGACTTCTTCTCAACTTGTAGGAGTT
2	20:43952512-43952534	TCGACCATCTGGAACCTTGC TGG	4	+	Intronic	TOX2	chr20:43952461-43952511	GGGTTGGGCTCTGCTCCACAGATCACTCAGGGCTGTAGGTTGGTCAAGTC	chr20:43952574-43952624	ATACTTCTACCCAGAGGAGACCCACGTTGTTTCTGCTCATAACCCATTGG
12	16:113736-113757	CCCTCCTGTTTATCTGAGA NGG	0	+	On-target	MCS-R2	chr16:113685-113735	AGGGAAAGGGGTGAATGGTACTGCTGATTACAACCTCTGGTGCTGCCTCC	chr16:113787-113837	CTTCAGGGGCAAAGCCTGACCCAGACAGTAAATACGTTCTTCATCTGGAG
12	10:121915516-121915538	CCCCTCCAGTTTGTCTGTGA TGG	4	+	Intronic	ATE1	chr10:121915465-121915515	ATTCCCCTGAACTAAAGGATAAGATGGGACGAGCCCCCAGATAAGAAGGG	-	-
12	11:1398795-1398817	TCCCTCCTGCTCATCTGTGA TGG	4	+	Intronic	BRSK2	chr11:1398744-1398794	GCAGGCCCTGCCGGTGTGGCTTCAGGAGTCCTGGTCCCCGCACTCAAGCT	chr11:1398817-1398867	GGCCTGGGTGTACCCAGGTCCTTGGTAGGCGCCAGGAGATGTGTGGGGCC
12	11:115380458-115380480	ACCCTAATGATTATCTGAGA TGG	4	+	Intronic	CADM1	chr11:115380407-115380457	GATCCCATTAAACCATCTCTGGGTACGCTATCCAGCTGAATGAAAATGAA	-	-
12	18:48673045-48673067	TCCATCCTGTGCATCTGAGA GGG	4	-	Intronic	CTIF	chr18:48672954-48673004	TTTCTCACCAGCAGGCGGTTCCCTCTGCCTAAAATCTCACTCAGCCCTTC	-	-
12	3:60409075-60409097	ACTCTCCTGTTTTGCTGAGA AGG	4	-	Intronic	FHIT	chr3:60409024-60409074	GAGTTGGGTGACTCCTGCAAGTTCCTTACTATTTATTTGATCCTCAGTCT	chr3:60409097-60409147	ACAGATTTCATTGGATTCTTGTAAATACTGAATATGATAGTGTATGTTAC
12	9:124886773-124886795	CACCTCCTGCTTCTCTGAGA AGG	4	-	Intronic	GOLGA1	chr9:124886682-124886732	AAAAAACGACCTAGGCAAGAAGGGGGAGGCGGCTGGCGTAGTGCTTGGGG	chr9:124886835-124886885	GGAGAGAAGGTAGACATGAAGTTGGGGTTAGGGGCAGAGGGGCGTGAAGG
12	7:44963946-44963968	TCTCCCCTGTTTACCTGAGA TGG	4	-	Intronic	MYO1G	chr7:44963895-44963945	AGGATTTTCAGCACGGGTGCCACCATCGCTGAGTTTTAGGATGGTCTGGG	chr7:44964008-44964058	TCACAGATGCTGCACCCTACTCCCCACCCACATTGCTCACCGCCTCAAGG
12	7:108238683-108238705	TCCCTCCTGTGTATCTGTCA GGG	4	+	Intronic	NRCAM	chr7:108238592-108238642	TCCCTCTGCTTGACAGATTGAACAGTCAGATTAAGGGGGAATATATTTAA	chr7:108238705-108238755	CAAATGCTTACTTAGGCATTCCCACTCCAATGGGCTTAAGCAATTGTATA
12	14:78597901-78597923	CCCTTCCTGTCTTTCTGAGA TGG	4	-	Intronic	NRXN3	chr14:78597850-78597900	AAGATGCACGTGATGACCACGGCTAGTTGATCCCTCTTGGCATATTGTTG	chr14:78597923-78597973	TGGGTTACAGCTGCCAGCACCATCAGGATTAAAAGCAGGAGGACTTTAAG
12	11:131549252-131549274	GACCTTGTGTTTATCTGAGA TGG	4	+	Intronic	NTM	chr11:131549201-131549251	TTGACAGCAGAAGCATGGAGGCATAGAAGCTGAGGCCACTATTGGGAAGG	chr11:131549314-131549364	GTCAGCAAAAATTATGTCTAGGGTCACACCCTATATAGAAGAAACCCCAG
12	20:9120242-9120264	TCCCTCCTTTTAATCTGACA TGG	4	-	Intronic	PLCB4	-	-	chr20:9120264-9120314	TTGGGAACAGTTCAAGGCCCTCTCTGAATGTCTTATTCAAATTCCCATTT
12	22:35822072-35822094	TCCCTCCTGCTTTCCTGAGA TGG	4	+	Intronic	RBFOX2	chr22:35822021-35822071	GGCCTATTACAAAGAGAGGGCCAGCCTTTCTGCCTTGTCTCACTCTTCTT	-	-
12	9:4854138-4854160	GACCTCTTTTTTATCTGAGA AGG	4	-	Intronic	RCL1	chr9:4854087-4854137	TTTGAGCCCTAGAACATAATTGGTGTATGGTAAAAGCAAGTGATAGCCTT	chr9:4854200-4854250	GGGACCCCATCTCCTGACAGTGCAGCTAGCCTAGTTCTCTGTTTCCTGCT
12	8:20151479-20151501	GCCCTTCTATTTCTCTGAGA GGG	4	+	Intronic	SLC18A1	chr8:20151428-20151478	ATACCCATACATAATTCCTGGGAAGTGAATTCCTGCCTTGCTACTTACCA	-	-
12	19:38271812-38271834	GCCCTCTTTTTTTTCTGAGA TGG	4	-	Intronic	SPINT2	-	-	chr19:38271834-38271884	AAGAATTCACATGTTGTTTTACTCACACGTGTTGGAGATGGAGCTCAGGG
12	3:63291375-63291397	CCCCTCCACTTTATCAGAGA GGG	4	+	Intronic	SYNPR	-	-	chr3:63291397-63291447	CTCGGTAAAGGTTGACTGTCCTCAAACCCAGAAAATTAAAGCTCAGATAT
12	18:31916503-31916525	AACCTCCTGTTTATCTCAGT AGG	4	-	Intronic	TRAPPC8	chr18:31916452-31916502	AACCATATTATTAAGGTAATTATCGCTTATTACTCAATGATAAATATTGT	-	-
1	16:113491-113512	ATAGGTTCCAGAGGGTCGA GGG	0	-	On-target	MCS-R2	-	-	chr16:113512-113562	CAGGGACCACAGTCAGCCAGGCAAGCACATCTGCCCAAGCCAAGGGTGGA
1	11: 7504765-7504787	GATGGGTTCCAGAGGTTCGG AGG	3	-	intronic	OLFML1	chr11:7504714-7504764	CTGCCCACACAGCTGGCATATAGCAAACAGTTTGCTAACATGCTTGCTTA	-	-
1	7: 31915080-31915102	GATTGGTTCCAGAGGGATGA TGG	3	-	intronic	PDE1C	chr7:31914989-31915039	GGTAAGGTATCTGAAAGTGCTGAACACACCGCCTGATGTAAAGCAGGTAT	chr7:31915102-31915152	CATTTGCTACCCAGGATAGAATTTAAGGTACTTTAAGTATCTAATCGCAA
1	2: 69752276-69752298	GACAGGTCCTAGAGGGTCCA AGG	4	+	intronic	ANXA4	chr2:69752225-69752275	CTAAGTCCATTCTGTAGGTGTATGGGAGACAGGGCAGATGAGTATTGAGG	chr2:69752298-69752348	ATCCCATGTAAGGAACTGCTGGCCTGGAATATAAATCCCTACTTCCCTTT
1	12: 2496273-2496295	GATAGTTTCCACAGGGAGGA GGG	4	+	intronic	CACNA1C	chr12:2496192-2496242	AGTTTGAAAACGCTGGAAATCTAACCTGTTGAACCTCATGGTGCTGGTAT	chr12:2496295-2496345	GGAAATAAGGCCTTTCACTCAGAAGAATGAATTGTCCTTCAGGCACAAGG
1	10: 12715111-12715133	GATAGATACCAGAGGCTGGA AGG	4	-	intronic	CAMK1D	chr10:12715060-12715110	GAACTGTATGTCTGTATCCACTCACCAATCCCCTCAGACGCCCCCTACAC	chr10:12715163-12715213	TTCTCAGCTACCACGTATGAGTCGTCACAGTTCTTGATGATATATTTCCC
1	19: 46009930-46009952	GGTTGGTTCCAAAGGGTGGA GGG	4	-	intronic	CCDC61	-	-	chr19:46009952-46010002	ACAGGAGCCTTGAGATCCCACCGCTGCTCAGTGGCTTCTTATCTTCCTCT
1	5: 19987948-19987970	GATGGGTTTCACADDATCGA GGG	4	-	intronic	CDH18	chr5:19987897-19987947	ATGTATACCAGCGATAAGGCAGCCTGCATTAGCTACAACTTTCCCCTCAG	chr5:19987970-19988020	GGAGATGAAGATTTCACCCAATATTGCTTTGACTCAGAACTGGGTAAAGC
1	10: 71737918-71737940	GCTTGGTTCCAGTGGGTAGA AGG	4	-	intronic	CDH23	chr10:71737867-71737917	TGCCCGGTTTTTATTTGACAGTCAGAAATTCACATGCCTTCCCCATCTGC	chr10:71737980-71738030	CCTCTAAGCCCAAGGTTGCAAACAAAGACACCTTCGGGCAATATCTGGCC
1	3: 128396586-128396608	GACTGGTTCCTGAGGGTGGA TGG	4	+	intronic	EEFSEC	chr3:128396495-128396545	GCACTGGGACACACTGTCAAATGGGATCGTCTCCGCCCTCCCAAAGCCCC	chr3:128396608-128396658	CCTACTGGGCATGAGGCTGTGGTGCTGTGAAGGGTTTGAGCTCAGGCCCA
1	7: 133962519-133962541	GAAGGGAACCAGAGGGTCGA AGG	4	-	intronic	EXOC4	chr7:133962468-133962518	AAAGATGATCATTGGGGCTACATAGCTTCAGCCCGGGCAGAGATGTGCCA	-	-
1	15: 65328877-65328899	GGCAGGCTCCAGAGGGTGGA CGG	4	-	exonic	IGDCC3	chr15:65328826-65328876	TCCCACATGACAGTAGAAATCTTGCTTTGACCTGAGAATGGGCCCCGCT	chr15:65328899-65328949	AGACACTGGCTACTGTTCCGAGTGAGCTGGCTGGGTAACCCGGGCCGCTG
1	12: 116475-116497	AATAGGTTCCAAAGGGACCA CGG	4	-	intronic	IQSEC3	chr12:116424-116474	GCATCCCCGGCATTTCCGTGAGCTATTCTTTTGTGGTTCCTGATGGTCAC	chr12:116497-116547	GCTTACTCAGGCCACAAAGGGCTGGGGTGTAGAGGGCTTAAGGAGTAACT
1	20: 43524569-43524591	GAAAGGTTCCATGGGGTAGA TGG	4	+	intronic	L3MBTL1	chr20:43524518-43524568	ATGTAAATACAAGGGGCTGTCACAGAACAAACTCTGCCTGCTGGAGTATA	-	-
1	14: 64394274-64394296	GGTAGGTGGCAGAGGCTCGA TGG	4	+	intronic	MTHFD1	chr14:64394183-64394233	TCCTCAGCCACTCGGCTACTACCCTTACCCTCAATTTTGCAGTTTATGAC	chr14:64394296-64394346	TGCATTTCTTTCCCTACCATGGGCTTGTCATTGGCTTGCTAGAAGGAATT
1	15: 88025647-88025669	TATAGGTTTCAGAGGGAGGA TGG	4	+	intronic	NTRK3	-	-	-	-
1	1: 2,520,380 to 2,520,402	GACAGGTTCGAGTGGGTCGG CGG	4	-	exonic	PANK4	chr1:2520329-2520379	TGGTGAGCAGAGCGCCAAGCCCCCAGAAGGTGCCGCCTCCAATGGAGCTG	chr1:2520402-2520452	CTCCGTCTCCACCTGCAACAGAGCCAGGGCAGGTGTGCCCTCAGTGGGCC
1	5: 131487303-131487325	CAAAGCTTCCAGAGGGTGGA AGG	4	+	intronic	RAPGEF6	-	-	-	-
1	12: 131742291-131742313	AATAGGTTCCAGAGAGTTGT GGG	4	-	intronic	SFSWAP	chr12:131742240-131742290	TTCTTTCCATGGTGGGGCCCAGGTCTCACTTCAGCCACTTGCTCTCTTTA	chr12:131742313-131742363	TTTATGTAAGAAGTCTTCAAAACCTCAGTACAGCATTAAAAATTGAAAGC
1	17: 9573003-9573025	TTTAGGTTCCAGAAGGTGGA GGG	4	+	intronic	STX8	chr17:9572952-9573002	TGTCCCTTATCTTTGACCTTTATTTCCTACTTGATTTGTAAACTGAGGGA	-	-
1	3: 18144885-18144907	TATAGGGTCCAGAGGCTGGA GGG	4	-	intronic	TBC1D5	chr3:18144804-18144854	CATGAGTTTGGTTATCCTCCTGGTCTCTTTACTGGTTCCAAAGGGAGGCT	chr3:18144907-18144957	CCCTCCCTACACAACCTTCTCTGAACTCTCTCTAAGGTCAGAAAAGGCAG
1	17: 78027622-78027644	GATAGGATCCAGAGGTTTGG AGG	4	+	intronic	TNRC6C	chr17:78027571-78027621	ACTTTGGAGAGAGTTGTGAGTTGAAGACCCAGGACACGCAAAGTCCCTAA	chr17:78027644-78027694	GTGCCCCCTTGCATTGCCTCTCTGACCCCATTGATTGTGAGACATATCGG
1	22: 28005714-28005736	GATAGGTGCCAGGGAGTCCA GGG	4	-	intronic	TTC28	chr22:28005633-28005683	AGCGCTGTCCCGTTGCCACCTTGCAGTCTCGGCCTGGTGATAATCTTCCC	chr22:28005736-28005786	ACCGCTGGAAGCTGATGGCTGCTCTCCTGCCCACACTCAGGGGGCTAGCG
1	8: 10973474-10973496	AATAGGTTTCAGAGGGTGGT TGG	4	-	intronic	XKR6	chr8:10973423-10973473	CTCCGTGTGCACCAGCCAGTCTTTCCCATACATTCTTTCATTCAATCTTC	-	-
1	10: 79168076-79168098	AATTGGCTCCAGAGGGTAGA GGG	4	-	intronic	ZMIZ1	chr10:79168025-79168075	GTGGGGTGTTTCTTAGGAGTCAGGCTGGCGGCAGAACAGCCCCTCCAGAG	chr10:79168098-79168148	TTCCTCTTTGTTACCTGCAAGCCCCCTTCAGCTGCACTTGGCTGTATGCT
8	16:113802-113823	CGTATTTACTGTCTGGGTC NGG	0	+	On-target	MCS-R2	chr16:113711-113761	ATTACAACCTCTGGTGCTGCCTCCCCCTCCTGTTTATCTGAGAGGGAAGG	chr16:113863-113913	AGATCCCATGTTCACAAACAGAACTCACACCTGGATGGACTTACAACAAG
8	7:129318890-129318912	TCGAATTTACTGTCTGGCTC AGG	3	-	Intronic	AHCYL2	-	-	-	-
8	12:2696698-2696720	TCTTATTCACTGTTTGGGTC TGG	4	+	Intronic	CACNA1C	chr12:2696647-2696697	TAAGAGAGGGTCATTTCTATGTGAGGAAATGCAGAAATGGACAGAATGAT	chr12:2696720-2696770	AGAATTCCCATTGTGGAAATCTTAGAGATCTCAAGTTTATTACCAAGGGA
8	17:66977851-66977873	GGGTATTTACTCTCTGGTTC TGG	4	+	Intronic	CACNG4	chr17:66977800-66977850	TGCTGAGTGCCTGGCTTCAGGCCGGGCATTGTGGGCACAGAAAGCCCCCA	chr17:66977873-66977923	AGACCCTGGGGCACCAGCAGTAGGACAGACACCATACAGGGCACGATGTG
8	15:42726967-42726989	AAATATTTACTGTCTGGTTC TGG	4	-	Intronic	CDAN1	chr15:42726916-42726966	GTACCTTATAGTCCCTATTCCAATCTCTATTCCTAGATCATTACCCTAGA	-	-
8	4:808708-808730	TCTTATTTCCTGTCTGGGTG TGG	4	-	Intronic	CPLX1	chr4:808657-808707	CAGAAAGAAGCCTGGGACACACAAAACGAGGGGAAACGATTGAGAACTAG	-	-
8	2:10311037-10311059	CCGTATTTACTGTGTGTGGC TGG	4	+	Intronic	HPCAL1	chr2:10310946-10310996	CCCTGTAATCAATTTTTGGAGAGAAGTGAGAACCTCTCGAAATCGATCAC	chr2:10311059-10311109	AGACACTCCTGGCAGAATGAGTGTGGGATGTAGGAGGTCCAGAAAAGCCC
8	16:47355284-47355306	TGGTATTTATTTTCTGGGTC TGG	4	-	Intronic	ITFG1	-	-	-	-
8	16:88775046-88775068	TCGTCTTCACTGTCTGGGCC GGG	4	-	Intronic	PIEZO1	chr16:88774995-88775045	TTGCAGGGACCCAAAGGCACCCACTCCATCCAGCAGACACCCAGGGAGGC	chr16:88775068-88775118	CAGAGTTCCCGGCCGTACCCGTGACACCCAGGACGTAACGGAGTGAGGGG

Supplementary table 4 – Results of off-target screening

CRISPR Number	Off-target location	Mis-matches	Strand	Feature	Gene	SNP/indel locations	Alleles (ref/alt)	MAF	Number in figure	Name in literature
1	chr1:2520380-2520402	4	-	exonic	PANK4					
1	chr2:69752276-69752298	4	+	intronic	ANXA4	chr2:69752263	A/T	0.2	17	rs6736776
1	chr3:128396586-128396608	4	+	intronic	EEFSEC	chr3:128396538	A/G[1]	0.2	13	rs1735527
1	chr3:18144885-18144907	4	-	intronic	TBC1D5					
1	chr5:19987948-19987970	4	-	intronic	CDH18					
1	chr7:31915080-31915102	3	-	intronic	PDE1C	chr7:31915036	G/T	0.3	15	rs1016191
						chr7:31915071	T/A	0.3	18	rs2109836
1	chr7:133962519-133962541	4	-	intronic	EXOC4					
1	chr8:10973474-10973496	4	-	intronic	XKR6					
1	chr10:12715111-12715133	4	-	intronic	CAMK1D					
1	chr10:71737918-71737940	4	-	intronic	CDH23					
1	chr10:79168076-79168098	4	-	intronic	ZMIZ1					
1	chr11:7504765-7504787	3	-	intronic	OLFML1					
1	chr12:2496273-2496295	4	+	intronic	CACNA1C	chr12:2496220	G/A	0.4	11	rs1015287
1	chr12:116475-116497	4	-	intronic	IQSEC3	chr12:116448	T/C	0.4	16	rs10744726
						chr12:116489	G/A	0.2	21	rs11064561
						chr12:116533	G/A	0.2	23	rs9668025
1	chr12:131742291-131742313	4	-	intronic	SFSWAP					
1	chr14:64394274-64394296	4	+	intronic	MTHFD1	chr14:64394292	G/C[2]	0.2	22	rs55739639
1	chr15:65328877-65328899	4	-	exonic	IGDCC3	chr15:65328800	G/A	0.4	10	rs11853777
1	chr17:9573003-9573025	4	+	intronic	STX8	chr17:9573081	C/del1	0.1	24	rs71135994
1	chr17:78027622-78027644	4	+	intronic	TNRC6C					
1	chr19:46009930-46009952	4	-	intronic	CCDC61	chr19:46009872-86	NA[3]	-	14	
1	chr19:46009930-46009952	4	-	intronic	CCDC61	chr19:46009925	NA[4]	-	19	
1	chr20:43524569-43524591	4	+	intronic	L3MBTL1					
1	chr22:28005714-28005736	4	-	intronic	TTC28					

8	chr2:10311037-10311059	4	+	Intronic	HPCAL1	chr2:10311039	A/G	0.3	20	rs16856098
						chr2:10310985	A/G	0.3	12	rs11695971
8	chr4:808708-808730	4	-	Intronic	CPLX1		-			
8	chr12:2696698-2696720	4	+	Intronic	CACNA1C		-			
8	chr15:42726967-42726989	4	-	Intronic	CDAN1		-			
8	chr16:88775046-88775068	4	-	Intronic	PIEZO1		-			
8	chr17:66977851-66977873	4	+	Intronic	CACNG4		-			
2	chr1:18294783-18294805	4	+	Intronic	IGSF21					
2	chr4:86840217-86840239	4	+	Intronic	SLC10A6					
2	chr5:139259569-139259591	4	+	Intronic	SIL1					
2	chr12:132908486-132908508	4	-	Intronic	CHFR	chr12:132908470	C/T	0.7	1	rs4758916
						chr12:132908532	A/G	0.2	5	rs4758917
2	chr14:104936545-104936567	4	+	Exonic	PLD4	chr14:104936657	T/C	0.4	8	rs10083374
2	chr20:43952512-43952534	4	+	Intronic	TOX2		-			
2	chr22:41697129-41697151	4	-	Exonic	C22orf46		-			
2	chrX:123429476-123429498	4	+	Intronic	GRIA3		-			
12	chr3:60409075-60409097	4	-	Intronic	FHIT		-			
12	chr3:63291375-63291397	4	+	Intronic	SYNPR	chr3:63291390-93	AGAG/del4	0.4	3	rs34549935
12	chr7:44963946-44963968	4	-	Intronic	MYO1G		-			
12	chr7:108238683-108238705	4	+	Intronic	NRCAM		-			
12	chr8:20151479-20151501	4	+	Intronic	SLC18A1		-			
12	chr9:124886773-124886795	4	-	Intronic	GOLGA1		-			
12	chr9:4854138-4854160	4	-	Intronic	RCL1	chr9:4854253	C/T	0.05	9	rs10974815
12	chr10:121915516-121915538	4	+	Intronic	ATE1	chr10:121915615	C/T	0.5	7	rs7893846
12	chr11:1398795-1398817	4	+	Intronic	BRSK2	chr11:131549255	C/T[5]	0.003	2	rs577118723
12	chr11:115380458-115380480	4	+	Intronic	CADM1	chr11:115380472	C/T	0.01	4	rs11608105
12	chr11:131549252-131549274	4	+	Intronic	NTM		-			
12	chr14:78597901-78597923	4	-	Intronic	NRXN3		-			
12	chr18:48673045-48673067	4	-	Intronic	CTIF		-			
12	chr18:31916503-31916525	4	-	Intronic	TRAPPC8	chr18:31916593	C/T	0.01	6	rs62093870
12	chr19:38271812-38271834	4	-	Intronic	SPINT2		-			

12	chr20:9120242-9120264	4	-	Intronic	PLCB4		-			
12	chr22:35822072-35822094	4	+	Intronic	RBFOX2		-			

1 Previously described SNP - G seen only in control sample

2 Variable site with novel SNP = G/C in hg38/hg38alt, G/T observed in both condition and control

3 Novel 15bp deletion - observed in both condition and control, not described in 1000 genomes database (phase1 Aug 2015)

4 Novel 1bp deletion - observed in both in condition and control, not described in 1000 genomes database (phase1 Aug 2015)

5 Previously described rare SNP - T seen only in condition sample

Supplementary table 5 – Clinical and molecular characteristics of the donors with HbE/β-thalassemia

	Donor 1	Donor 2	Donor 3
Age	33 years	30 years	13 years
Sex	Female	Female	Male
Clinical Phenotype	Moderate-severe HbE/beta-thalassaemia	Moderate-severe HbE/beta-thalassaemia	Moderate-severe HbE/beta-thalassaemia
Beta-thalassemia mutation	IVSI-5 G>C	IVSI-5 G>C	IVSI-5 G>C
Alpha globin status	αα/αα	αα/αα	αα/αα
Age at first blood transfusion	3 years	8 months	6 years
Hemoglobin before first blood transfusion (g/dl)	Not known	8.7	4.8
Pre transfusion HPLC			
HbA (%)	19.2	16.9	4.9
HbF (%)	38.3	39.8	30.6
HbE (%)	39.5	42.8	60.5
Spleen	Splenectomy at 9 years	Splenectomy at 13 years	Spleen - 10cm
Transfusion requirement	3-4 monthly	3-4 monthly	2-3 monthly
Serum Ferritin (ng/ml)	846	374	672
